# Can Saccade and Vergence Properties Discriminate Stroke Survivors from Individuals with Other Pathologies? A Machine Learning Approach

**DOI:** 10.3390/brainsci15030230

**Published:** 2025-02-22

**Authors:** Alae Eddine El Hmimdi, Zoï Kapoula

**Affiliations:** 1 Orasis-Eye Analytics & Rehabilitation Research Group, Spinoff CNRS, 12 Rue Lacretelle, 75015 Paris, France; 2 LIPADE, French University Institute Laboratoire d’Informatique Paris Descartes, University of Paris, 45 Rue des Saints Pères, 75006 Paris, France

**Keywords:** saccade, vergence, stroke, developmental disorders, machine learning

## Abstract

Recent studies applying machine learning (ML) to saccade and vergence eye movements have demonstrated the ability to distinguish individuals with dyslexia, learning disorders, or attention disorders from healthy individuals or those with other pathologies. Stroke patients are known to exhibit visual deficits and eye movement disorders. This study focused on saccade and vergence measurements using REMOBI technology V3 and the Pupil Core eye tracker. Eye movement data were automatically analyzed with the AIDEAL V3 (Artificial Intelligence Eye Movement Analysis) cloud software developed by Orasis-Ear. This software computes multiple parameters for each type of eye movement, including the latency, accuracy, velocity, duration, and disconjugacy. Three ML models (logistic regression, support vector machine, random forest) were applied to the saccade and vergence eye movement features provided by AIDEAL to identify stroke patients from other groups: a population of children with learning disorders and a population with a broader spectrum of dysfunctions or pathologies (including children and adults). The different classifiers achieved macro F1 scores of up to 75.9% in identifying stroke patients based on the saccade and vergence parameters. An additional ML analysis using age-matched groups of stroke patients and adults or seniors reduced the influence of large age differences. This analysis resulted in even higher F1 scores across all three ML models, as the comparison group predominantly included healthy individuals, including some with presbycusis. In conclusion, ML applied to saccade and vergence eye movement parameters, as measured by the REMOBI and AIDEAL technology, is a sensitive method for the detection of stroke-related sequelae. This approach could be further developed as a clinical tool to evaluate recovery, compensation, and the evolution of neurological deficits in stroke patients.

## 1. Introduction

Ocular movements, whether voluntary (saccades, vergence, pursuit, fixation) or reflexive (vestibulo-ocular, optokinetic), are crucial for vision. The neurological bases of these movements are well understood in human studies using transcranial magnetic stimulation (TMS) or magnetoencephalography (MEG) [1,2,3]. Moreover, the study of their parameters (latency, precision, speed, binocular coordination) provides valuable insights into brain function at various levels.

Beyond vision, ocular movements play a significant role in guiding and planning motor actions, as well as controlling balance and postural stability [4]. Ocular movements are intertwined with executive cognitive functions [5,6,7]; for instance, the saccade latency was found to be correlated with the Mini-Mental State Examination (MMSE) score [7]. Moreover, eye movements are vital for gaze and social interaction, making them a window into the brain.

The present study focuses on saccades (rapid eye movements of the two eyes in the same direction to fixate, i.e., via the foveas, on an object in the perifoveal or peripheral field) and convergence or divergence eye movements in opposite directions in the two eyes, enabling one to increase the convergence angle of the optic axes to fixate on a nearby object or to decrease it to fixate on a faraway object (divergence). Here, we emphasize the link between saccades and vergence in terms of their spatiotemporal properties, attention, perception, motor control, and executive functions. For instance, the fact that the latency or preparation time is prolonged implies that all subprocesses that underlie eye movement programming, such as shifting one’s attention and perception to localize a target in the periphery, computing the correct motor command, and deciding on the moment at which to trigger a movement, are altered in stroke patients. The series of hypothetical subprocesses occurring in parallel in the human brain during eye movement preparation has been presented by Findlay and Walker. Moreover, the saccade or vergence latency indicates the time required to prepare the eye movement command and reflects brain functioning, being generally longer in young children (less than 7 years old) and in seniors (beyond 60 years old) than in younger adults. When the cerebral parietal–frontal circuits involved in saccade preparation are affected, the latency can become longer [8]; note that other parameters, such as the amplitude and velocity, of the eye movements are less dependent on age as they are governed more by the subcortical eye movement generators in the brainstem.

Additionally, eye movements and attention shifts are closely linked. The theory introduced by Rizzolatti in 1987 [9], known as the “premotor theory of visual attention”, suggests that any attention shift involves an eye movement command, i.e., of a saccade, regardless of whether the saccade is executed.

In neurodevelopmental disorders such as dyslexia, reading difficulties, and learning problems, attention deficits are frequently implicated. In the past, saccade abnormalities, particularly latency issues, have been reported in such populations [10]. Other studies have indicated abnormalities in the binocular coordination of saccades, but also in the velocity of saccades [11]. Abnormalities in metrics such as the saccade amplitude, accuracy, or precision may reflect issues at multiple levels, i.e., in the cortical and subcortical neural circuits controlling the programming and execution of eye movements.

In parallel, machine learning studies applied to the eye movement data of children with neurodevelopmental disorders have been developed recently to classify various pathologies. These studies will be briefly described next. In the context of screening for dyslexia using eye movement parameters, Rello et al. [12] used a dataset of 97 patients to train a support vector machine (SVM), achieving accuracy of 80.18%. Similarly, Benfatto [13] applied an SVM to a different dataset of 185 patients, reporting accuracy of 95.6%. Smyrnakis et al. [14], on the other hand, employed a Bayesian model on a dataset of 66 children, achieving a correct classification rate of 94.2%. Asvestopoulou et al. [15] experimented with multiple classifiers on a dataset of 66 subjects, reporting accuracies of up to 97%. Jothi Prabha [16] explored clustering using k-means on a dataset of 97 samples. Finally, Prabha et al. [17] applied particle swarm optimization with an SVM on a dataset of 185 subjects, achieving accuracy of up to 95%.

Overall, these results are promising; however, they are based on research data rather than clinical data, which often show low signal quality, strong variability in the recording protocols [18], and relatively small sample sizes. Additionally, the negative class is not representative of real-world model deployment. The negative class should ideally consist of both healthy individuals and individuals with other diagnoses, so as to better simulate the model’s inference conditions. When these constraints are addressed, the learning task becomes more challenging, but it reduces the biases in the evaluation process, thus enhancing the reliability of the findings [19,20,21,22,23].

Other studies have examined additional tasks using eye movement parameters and multiple classifiers, such as mind wandering detection [24], predicting emotional states [25], detecting cognitive interference [26], and estimating cognitive workloads [27]. On the other hand, several studies have explored an end-to-end approach to learning from eye movement data, using deep learning models for various tasks, including age and gender classification [28,29] and screening for conditions like autism spectrum disorder [30,31,32,33,34], dyslexia [35,36,37], Alzheimer’s [38,39], and Parkinson’s [40] disease, along with other classification and regression tasks [41,42,43,44]. Recent studies in our group using machine learning have shown that saccade abnormalities can predict dyslexia in adolescents with a success rate of 83% or higher [45]; these studies used laboratory datasets. Additional studies using larger sets of clinical data with an end-to-end approach and deep learning further confirm that eye movement abnormalities can be used to successfully identify individuals with learning disorders from other populations [34,35,36,37].

Taken together, this body of literature converges regarding the fact that children with learning problems, including dyslexia, exhibit saccade and vergence abnormalities at various levels, and this could interfere with attention and cognitive executive functions. Now, turning to stroke survivors, recent studies [46] indicate that 73% of stroke patients experience visual problems, with 56% having visual deficits, 40% exhibiting abnormal eye movements, 28% experiencing visual field loss, and 27% facing visual attention issues. Only 27% have normal orthoptic visual exams. Note that, in these studies, eye movements were examined clinically via orthoptic tests, and their precise latency, accuracy, velocity, and binocular coordination were not recorded. We hypothesize that, because the neural network underlying saccade and vergence eye movement programming and execution is complex and resource-intensive, any neurological event, such as a stroke, would likely affect one or more properties of eye movements such as saccades or vergence.

The purpose of the present study is to investigate whether machine learning algorithms applied to saccade and vergence eye movement parameters (e.g., latency, accuracy, velocity, etc.) in individuals with a history of stroke, whether recent or in the past, can differentiate them from other populations, such as children with dyslexia or learning disorders, or from a more general population encompassing a variety of pathologies. The rationale is as follows: children with neurodevelopmental attention problems may exhibit abnormal latencies and inaccurate eye movements. Conversely, in patients with stroke, brain injuries can affect the circuits responsible for eye movement programming, leading to abnormalities in parameters such as latency and accuracy. In other words, although the underlying causes differ, both populations may exhibit eye movement abnormalities due to the link between attention and eye movements.

This raises a key question: to what extent are these abnormalities distinct enough to enable differentiation between the two populations using machine learning algorithms? To address this, the present study conducts a second analysis of previously published data on children with learning disorders [18] and integrates data from a population of stroke patients who performed the same saccade and vergence tasks using identical protocols. The study also considers age differences between the groups. For example, the latency parameter of eye movements is known to be sensitive to age, particularly in children below 10–12 years of age. In this study, the mean age of the child population is higher (13.63 years), mitigating some concerns about age effects.

The second objective is to determine whether machine learning applied to eye movement parameters can be used to differentiate stroke patients from a larger mixed population, including individuals with vertigo, strabismus, listening or expressive difficulties, postural problems, presbycusis, vertigo-related headaches, visual fatigue, and optical correction issues and even healthy individuals. These mixed population data are extracted from the dataset of a previous study that explored the use of deep learning approaches to screen for learning disorders [18]. To further account for age differences, an additional analysis is performed, focusing on differentiating stroke patients from an age-matched group of adults and seniors undergoing physiological aging [47]. Some individuals in this group had presbycusis but no cognitive or attentional decline. All data were collected from multiple clinical centers, using the REMOBI and AIDEAL technologies.

To summarize, the present study aimed to screen and identify stroke patients among various other groups of children or adults, each of them presenting different causes of eye movement dysfunction. Providing such a multifaceted comparison is an advantage for machine learning, avoiding the usual biases in binary comparisons of dysfunctional versus healthy conditions. The comparison with children presenting learning and attention deficits allows us to determine whether neurodevelopmental attention disorders and neurological deficits of attention in adults lead to different eye movement disorders.

The findings demonstrate that eye movement parameters can be used to differentiate stroke patients from children with developmental attention problems and learning disorders; distinguish stroke patients from a general mixed population with diverse pathologies; and differentiate stroke patients even more effectively from an age-matched group of healthy adults and physiologically aging individuals.

## 2. Materials and Methods

### 2.1. Datasets

Eye movements were recorded across various clinical centers in France and Europe (approximately 20). The available information only included a stroke annotation and the age of the patient, with the age data not specifying the exact birth date so as to protect patients’ anonymity.

The first objective was to use the quantitative capabilities of machine learning applied to a number of parameters in the AIDEAL FR software to determine whether saccade and vergence tests performed with the REMOBI device can distinguish stroke patients from children with neurodevelopmental disorders. All recordings were labeled by clinicians using a checkbox labeled “Neurological Stroke”; no information was available on the stroke events, such as whether they were recent or occurred several years prior, making it particularly interesting to assess whether eye movement patterns could be used to identify stroke patients within this mixed population with limited clinical annotations.

The second objective of the study was to detect stroke patients from a larger global population including those with attention deficits, dyslexia, and reading or learning disorders, as well as other pathologies, such as strabismus, vertigo, etc., aiming to examine a more realistic scenario by including various types of non-stroke patients to form the negative class. This second evaluation dataset better represented the model’s inference conditions, as the model had to be able to identify stroke patients among a broader population.

**Figure A1:**
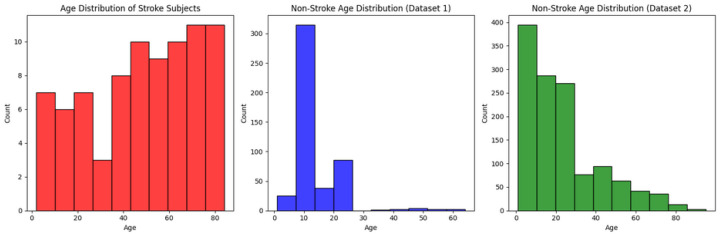
The age histograms of the population.

In Table 1, we present the characteristics of each population in terms of their age (years) and the mean and standard deviation of the age. Additionally, Figure A1 in Appendix A shows the age histograms for each of the three populations. The learning disorder population was the youngest, with a mean age of 13.63 years; the global clinical population presented a mean of 23.36 years; and the stroke population was more advanced in age, i.e., 48.45 years. The histograms in Figure A1 show that both the stroke population and the global population covered the entire age spectrum from 0 to 80, while the learning disorder population was more age-limited. To better account for age differences, we included a third group of adults (age-matched group), consisting of both younger adults and aging adults, some of whom presented presbyacusis in the absence of other pathologies. These data were originally published by [47].

### 2.2. Eye Movement Recording

**Figure A2:**
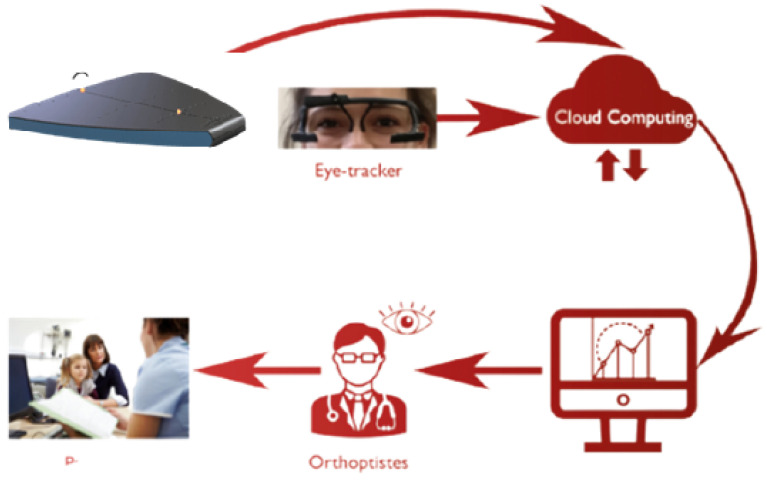
An overview of the entire setup used to record and analyze eye movements.

In all centers, eye movements were recorded using the same technologies, including the Pupil Core eye tracker. All clinical centers employed the same technologies, namely the REMOBI and AIDEAL version 3 technology by Orasis-EAr (www.orasis-ear.com), to stimulate and analyze eye movements. Figure A2 provides an overview of the setup used for the recording and analysis of eye movements.

REMOBI is a trapezoidal tablet upon which LEDs and buzzers are placed at four isovergence arcs from the patient’s eyes; paradigms are run that enable one to record and analyze the saccade and vergence eye movements in a 3D space.

Briefly, during the saccade task, the subject first fixated on an LED target at the center for a period of <1 s; then, the LED target appeared to the left or to the right pseudorandomly at eccentricity of 16 degrees. The central LED disappeared 200 ms later (overlap period), followed by the eccentric LED about 1 s later. The tests included 20 trials to the right and 20 trials to the left. Only centrifugal saccades towards the target were analyzed in the AIDEAL software. For the vergence test, the initial fixation LED was presented at 36 cm; then, after about 1 s, the target LED appeared either at 20 cm in the center or 100 cm away, calling for a convergence (8 degrees) or divergence eye movement (7.5 degrees); again, the fixation LED disappeared 200 ms later (overlap period). The test contained 40 trials, i.e., 20 for convergence and 20 for divergence, which were randomly implemented.

**Figure A3:**
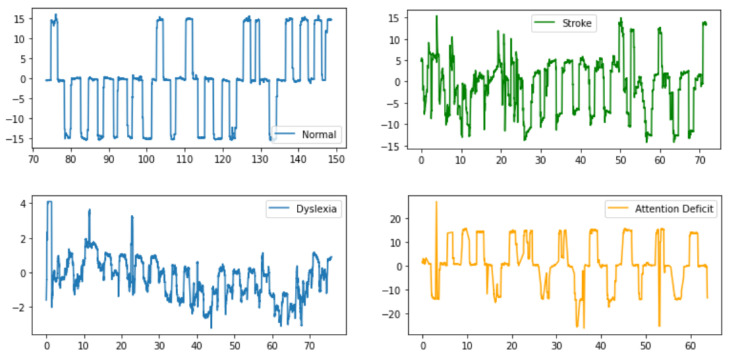
Representative examples of saccade time series (conjugate, i.e., mean of the left and right eyes) from a healthy individual, a stroke patient, a dyslexic individual, and an individual with an attention deficit. Stroke patients move the eyes predominantly to the left, indicating a right field omission; moreover, saccades to the left are performed with multiple staircase steps, rather with a single step, and this is in line with both the statistical data presented and the machine learning results. The dyslexic child shows similar behavior, saccading mostly to the left. For the individual with an attention deficit, saccades are oriented to either site but frequently with back-and-forth eye movements, suggesting difficulty in maintaining stable fixation.

**Figure A4:**
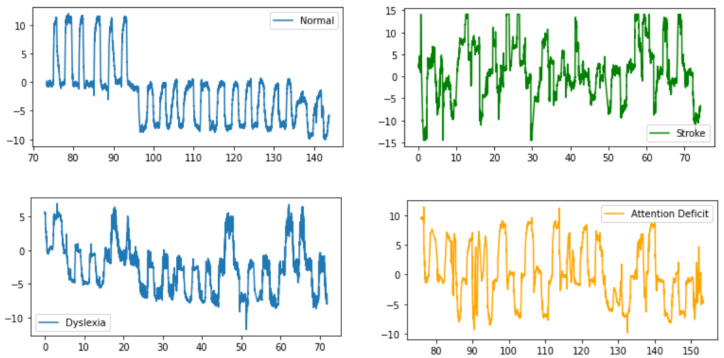
Representative examples of vergence eye movement time series (disconjugate signal, i.e. left–right eye difference) from a healthy individual, a stroke patient, dyslexic individual, and an individual with an attention deficit. The vergence from the stroke patient, particularly convergence, is higher but unstable, with many back-and-forth instabilities. The trace from the individual with an attention deficit shows similar behavior.

Time series data for two types of eye movement were examined: the horizontal saccade and vergence tests. Figure A3 and Figure A4 present examples of the conjugate time series (mean of the two eyes) for the saccades and disconjugate signals (difference between the two eyes) for the vergence eye movement tests.

The eye movement data series were sent by the clinician to the AIDEAL analysis platform in the cloud. This platform enables a quantitative analysis of many parameters of eye movement: the latency, amplitude, duration, maximal or average velocity (amplitude/duration), binocular coordination estimation, saccade disconjugacy (difference in amplitude of saccade between the two eyes), and disconjugate post-saccadic drift 1 and 2 at the first 80 and 160 ms after the offset of the saccade. These parameters were computed for rightward and leftward saccades, and the mean and standard deviation, together with the number of measures, were obtained. This enabled the complete characterization of the eye movement performance.

For vergence eye movements, the AIDEAL calculates the latency, the amplitude (the initial phasic component, i.e., the initial part lasting less than 80 ms from the onset of the movement or the amplitude after adding the movement during the next 80 ms or during the next 160 ms); the maximal or peak velocity, usually reached at the beginning of the movement; and the average velocity (amplitude/duration of the movement). All parameters were computed for convergence and divergence eye movements separately.

These individual means were then used for the statistical evaluation of the differences between any two groups and for machine learning.

### 2.3. Machine Learning Problem Formulation

Our dataset consists of *N* pairs, $D=(X_{i},y_{i})$, where each pair corresponds to a set of eye movement parameters, $X_{i}$, and their associated stroke annotations, $y_{i}$. The eye movement parameters are computed from each recording using the AIDEAL software, which enables the analysis of recordings based on the type of test performed (saccade or vergence). AIDEAL provides 32 parameters for the saccade dataset and 36 for the vergence dataset, including individual means and standard deviations. Our objective is to predict $y_{i}$ given the input $X_{i}$ using machine learning algorithms, thereby solving multiple binary classification problems.

We consider two ML analyses. In the first analysis, we explore the screening of stroke patients among individuals with learning disorders. In the second analysis, we move a step further by exploring stroke screening among a more variable group to mimic realistic clinical scenarios.

### 2.4. Data Pre-Processing

Each eye movement recording is analyzed using the eye movement analysis software AIDEA, which implements a velocity threshold criterion in order to detect the onset and offset of the movement; we then compute the different parameters presented in Table A2.

To address outliers, we apply different strategies tailored to statistical analysis and machine learning training. In the AIDEAL software analysis, non-physiological trials are filtered out by excluding values outside the confidence interval, before aggregating the trial parameter values by mean and standard deviation. When a recording contains at least five trials, these mean and standard deviation values are valid. If none of the values fall within the defined physiological limits (e.g., the latency is too short or too long, or the amplitude is too small or too large), the parameter values are set to 0.

In the machine learning analysis, we retain these 0 values, rather than replacing them with the mean of the entire training set, to preserve this information. In the statistical analysis, however, which involves analyzing the mean and standard deviation of each population, we filter out the 0 values before aggregating the remaining values by mean and standard deviation.

Table 2 presents the number of saccade and vergence tests recorded for each population, including data from stroke patients (dataset 1), individuals with learning disorders (dataset 1), and a broader clinical population (dataset 2). The number of recorded files is shown for both the saccade and vergence tests, along with the imbalance factor. This population imbalance was accounted for and corrected. In the first analysis, the problem was relatively well-conditioned compared to the second, particularly with respect to the imbalance factor (2.92 vs. 7.52 for the saccade dataset and 5.35 vs. 14.98 for the vergence dataset). However, the second analysis provides a more practical setup, as it better reflects real-world deployment conditions, where the model must identify stroke subjects not only among patients with learning disorders but also within the broader population.

### 2.5. Model Selection

For each of the two analyses, we train three different models: logistic regression, SVM with an RBF kernel, and random forest.

- Logistic Regression: This model establishes a linear decision boundary, making it suitable for linearly separable data. The boundary is constructed using a linear combination of the features, making it particularly well suited for small datasets.
- Support Vector Machine (SVM): Using a non-linear kernel, this model can create complex, non-linear decision boundaries by mapping the input features into a higher-dimensional space through the kernel trick, thus without explicitly transforming the data. It is effective for complex datasets but requires careful hyperparameter tuning.
- Random Forest: This ensemble method builds multiple decision trees and aggregates their predictions, allowing it to capture complex interactions and non-linear relationships.

Each of these three methods constructs its decision boundaries differently. Logistic regression creates a linear decision boundary through a logistic function based on a linear combination of features. In contrast, SVM with a non-linear kernel, such as RBF, maps the data into a higher-dimensional space to construct non-linear decision boundaries. Logistic regression operates in the primal space (using the eye movement parameter space), directly working with the input data to optimize the parameters. On the other hand, SVM with an RBF kernel primarily focuses on the dual space, optimizing the parameters related to the support vectors by leveraging the kernel trick to address non-linearities without explicitly mapping the data to a high-dimensional space.

We employed the scikit-learn implementation to fit all three algorithms. Given the relatively small dataset, it was impractical to split it into training, validation, and testing sets. Thus, we omitted the hyperparameter tuning phase and used the algorithms’ default parameters, which were mostly defined statically based on the characteristics of the fitted problem, such as the parameter size.

For all three algorithms, we utilized the balancing feature implemented within the scikit-learn library. Specifically, the balanced parameter allows the rescaling of the per-sample loss terms to mitigate the high imbalance factor.

### 2.6. Model Training and Evaluation

The models are trained using stratified cross-validation [48], implemented through the Sklearn library [49]. Cross-validation is an iterative process that maximizes the use of the dataset by splitting it into n_fold consecutive folds. In each iteration, (n_fold − 1) folds are allocated for training, while the remaining fold is reserved for evaluation. Stratified cross-validation is a variant that maintains the proportion of positive and negative samples in both the training and test sets.

For this study, the number of folds is set to 5. At the start of each fold, the data are normalized using the standard scaler from Sklearn [50]. The standard scaler adjusts each feature to have a zero mean and a standard division of 1; we also experimented with the MinMax implementation provided in the same library, but we observed similar performance.

Each training fold is standardized independently. During each fold of cross-validation, the data are standardized using only the training folds to prevent data leakage.

### 2.7. Threshold Decision Optimization

Our initial training setup resulted in a classifier with low sensitivity when using 0.5 as the threshold to convert the class probabilities into model decisions. To address this, we applied post hoc threshold tuning methods, which optimize the decision thresholds for classification models through cross-validation, using the balanced accuracy criterion to improve the performance.

We used TunedThresholdClassifierCV [51] from the scikit-learn library. To update the training pipeline, we wrapped the initial classifier with this object. During model fitting on the training set, inner cross-validation was performed exclusively on the training data, splitting them into training and validation subsets. In this process, the training folds are used to train the model, while the validation fold is used to adjust the threshold.

Initially, the model was trained with the default decision threshold set to 0.5. This means that samples with predicted probabilities above 0.5 were classified as positive, while those below 0.5 were classified as negative.

However, the decision threshold can be adjusted to improve the performance of specific metrics, although this may compromise others. In our case, we aimed to increase the sensitivity, accepting a minor reduction in specificity. The algorithm adjusts the model’s decision threshold to maximize the average sensitivity and specificity scores, which correspond to recall for the positive (stroke) and negative (non-stroke) classes, respectively.

## 3. Results

### 3.1. Statistical Evaluation of Group Mean Differences

#### Saccades

We ran a Kruskal–Wallis H test with independent samples to compare the group mean of the stroke patients with the group mean of the learning disorder population and with that of the global clinical group. This was performed for each of the eye movement parameters cited above and calculated using the AIDEAL software.

In Table 3, we show the group mean values and the Kruskal–Wallis test values with asterisks, indicating the level of significance; the results are shown for saccades to the right and to the left. The most statistically significant differences between the groups concern the latency, the amplitude, and the velocity: it is interesting that the stroke group shows, on average, longer latencies relative to each of the other two groups, as well as the smallest amplitudes and lower average velocities. This is the case for both rightward and leftward saccades. Thus, the classic statistical evaluation reveals delayed latency, smaller amplitudes, and lower average velocities in stroke patients.

Table 4 contains the data for vergence eye movements. For each parameter of convergence or divergence, we show the group means for each of the three groups, i.e., the stroke group, the learning disorder group, and the global clinical group, together with the values of the Kruskal–Wallis test, with asterisks indicating the level of statistical significance. The latency is again significantly longer for the stroke group than the global clinical population group; similarly, for divergence, the latencies are longer for the stroke group. The average and maximal velocities are higher for the stroke group than for the other two groups.

This observation is rather surprising; however, the scrutinization of the vergence eye movement traces indicates the instability of the vergence movement and strong contamination with saccadic intrusions (see Figure A4). This velocity increase could be the consequence of coupling vergence with saccadic intrusions, which enhance the vergence velocity, as there is a known acceleration effect between the vergence and the saccade [48]. The amplitude of the initial phasic part of the divergence is significantly larger for the group of stroke patients than for the other two groups.

To summarize, the latencies of both saccades and vergence eye movements are longer in stroke patients, the saccade amplitude is smaller, and the velocity is lower for saccades but higher for vergence, presumably due to saccadic intrusions. This analysis considering the eye movement parameters indicates physiological eye movement differences between these groups. Before presenting the ML approach, we next present a correlation analysis regarding the annotations of the patient by the clinician and using the eye movement parameters.

### 3.2. Screening Stroke Using Machine Learning

#### 3.2.1. Analysis 1: Screening Stroke Among Learning Disorders

Table 5 presents the three models’ performance in terms of the different metrics; the different results are aggregated by the mean on the test set of each fold, when training on the screening of stroke among learning disorder patients. Overall, all three models exhibit better performance on the saccade test data (60.0%, 70.2%, and 74.1%) compared to the vergence task (59.5%, 62.4%, and 77.2%). Moreover, the order of performance for the three models, when trained on each of the two tasks, is consistent across the four metrics.

When considering the performance of each model separately, the random forest model achieves the best overall performance, with a macro F1 score of 74.1% and 77.2%. In contrast, the best sensitivity is achieved by the SVM model on the saccade visual task (65.9%) and by the linear model on the vergence visual task (60.9%), while the best specificity is achieved with the random forest model on both tasks (96.5% and 99.5%).

**Figure A5:**
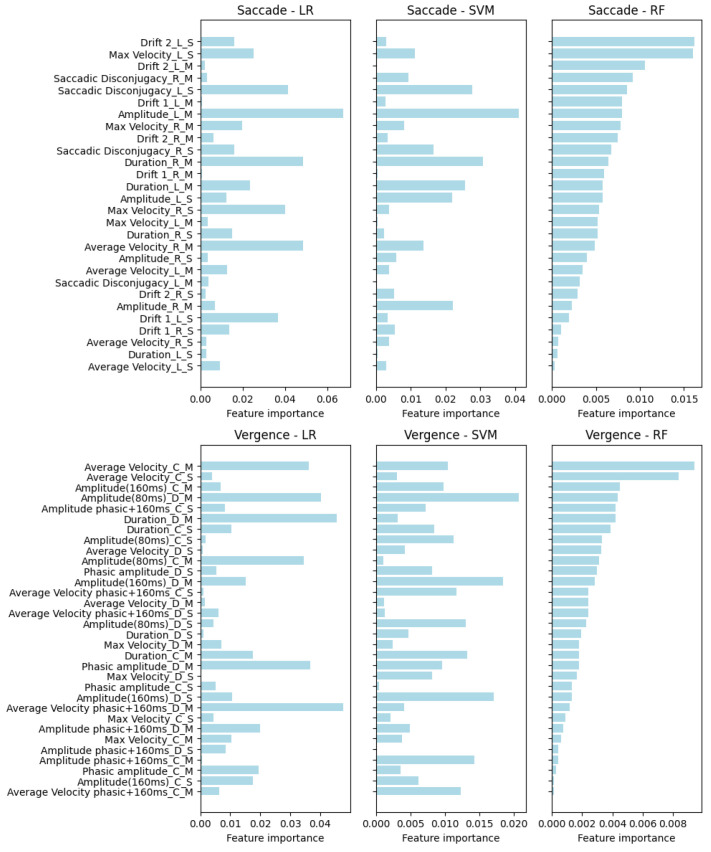
The feature importance for each dataset and for each algorithm when screening for stroke among learning disorders.

Additionally, Figure A5 presents the feature importance for each eye movement test and each dataset. Overall, for the saccade test, the latency and duration are the most determining features for all three models. On the other hand, the amplitude is not utilized by any of the three models, despite being correlated with the annotation.

In contrast, for the vergence test, each model uses a distinct set of features with varying levels of importance. The only shared important feature across the models is the latency. Additionally, when considering the best model in terms of the macro F1 score, the most relevant parameters are the average velocity and latency. These observations are in line with the initial statistical analysis using the Kruskal–Wallis test.

#### 3.2.2. Analysis 2: Screening Stroke Among Learning Disorders

In Table 6, we present the different models’ scores when trained on the second dataset, involving the screening of stroke among a global clinical population with other pathologies. Similarly, the random forest model obtained the highest F1 scores, with 75.6% and 77.1%, whereas the linear models showed the lowest scores, at 54.6% and 54.2%. Moreover, the best sensitivity was achieved by the linear model (48.5% and 75.2%), while the best specificity was achieved with the random forest (99.9% and 100%). Furthermore, the saccade dataset showed the highest performance relative to the vergence task.

While the random forest model achieves promising scores in terms of the macro F1 score, the performance when screening for stroke and non-stroke conditions is not the same, as the model achieves sensitivity of 38.3% and 39.0% on the saccade and vergence tasks, respectively, while it achieves the maximum specificity of 99.9% and 100.0%, suggesting that the model tends to favor the accuracy of the most representative class by exploiting the high imbalance ratio in the dataset to achieve a higher score. The result suggests that the loss reweighting technique only attenuates the imbalance effect on the training, without removing it completely.

Furthermore, we provide Table 7, which displays the different random forest scores before and after decision threshold optimization. Overall, the final scores demonstrate balanced performance in screening for both stroke and non-stroke subjects, as the model improves from sensitivity of 38.3% and specificity of 99.9% to balanced performance with sensitivity of 76.6% and specificity of 74.0%. Similarly, when considering the vergence dataset, the trade-off between sensitivity and specificity improves from 39.0% and 100.0% to 83.8% and 68.7%. Finally, in Figure A8 and Figure A9, we provide the confusion matrices before and after threshold tuning for each of the two tests (saccade and vergence).

**Figure 1:**
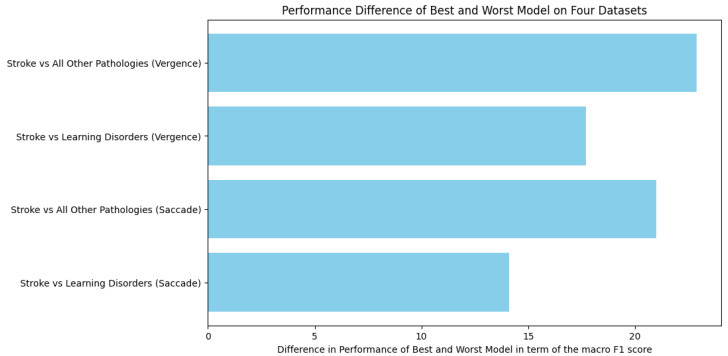
Barplot of the performance drops between the best and the worst models for each of the four datasets in terms of the macro F1 score. The benefit of using a complex approach is estimated using the difference, in terms of the macro F1 score, between the linear model and the random forest model.

Relative to the previous analysis, when comparing the performance differences between the best and worst models across the different datasets, as shown in Figure 1, the order of the three models in terms of overall performance (F1 score) remains consistent across both eye movement tasks.

**Figure A7:**
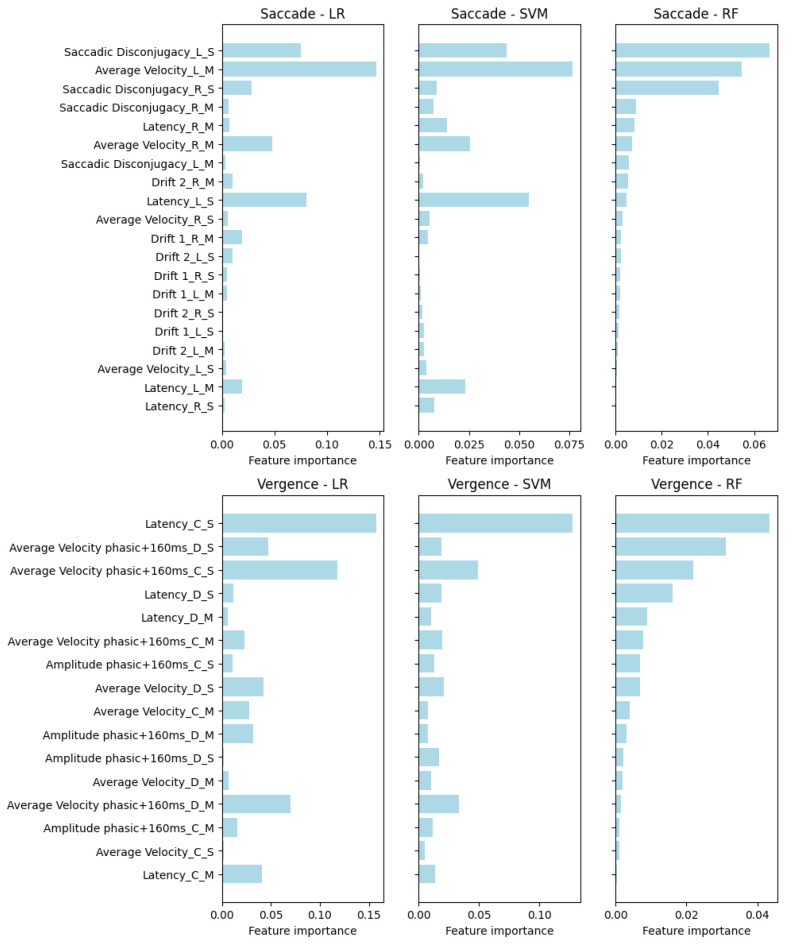
The feature importance for each dataset and for each algorithm when screening for stroke in the age-matched dataset in analysis 3.

**Figure A8:**
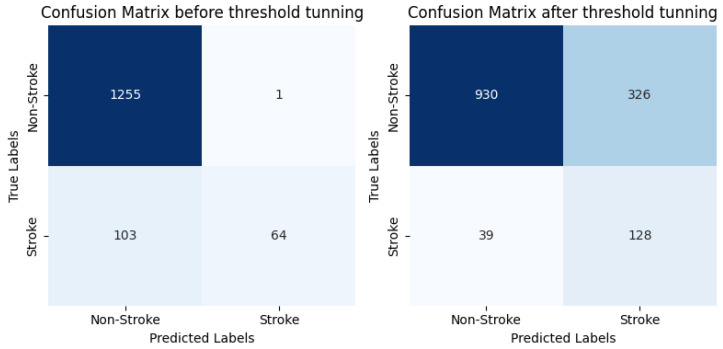
The confusion matrix when learning to screen the stroke population in the second analysis, on the saccade eye movement data, using random forest without threshold tuning (left subfigure) and with threshold tuning (right subfigure).

**Figure A9:**
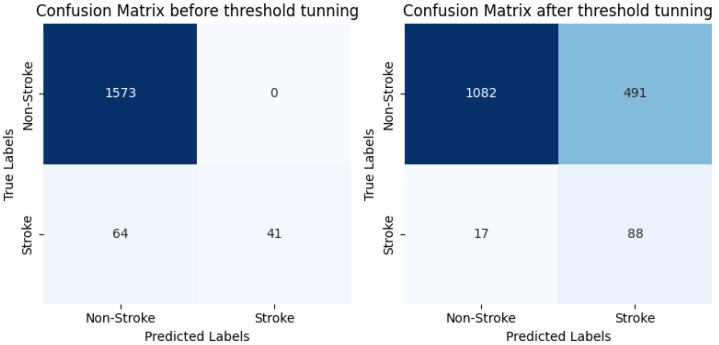
The confusion matrix when learning to screen the stroke population in the second analysis, on the vergence eye movement data, using random forest without threshold tuning (left subfigure) and with threshold tuning (right subfigure).

However, the difference in performance between the best and worst models increased, as shown in Figure A7. In the saccade task, the difference rose from 14.1 points to 21 points, and, in the vergence task, it increased from 17.7 points to 22.9 points, suggesting that the gap between linear and tree-based models scales with the task complexity.

### 3.3. Additional Analysis: Stroke vs. an Age-Matched Subgroup

In the final analysis, we considered a dataset of adults with an average age similar to that of the stroke group. Since the saccade amplitudes tested in the previous study were larger (20 degrees) compared to the amplitudes tested in the stroke group and other populations reported here (16 degrees), this analysis focused solely on eye movement parameters that were not dependent on the amplitude. In contrast, the vergence requirements are nearly identical across studies.

We present, in Table A4 and Table A3, the corresponding mean-based ANOVA comparison for the saccade and the vergence data, respectively.

The three machine learning models were applied to this dataset. The results are summarized in Table 8, with the barplot illustrating the most determinant eye movement parameters shown in Figure 1. The sensitivity, specificity, and F1 scores were high, demonstrating the models’ ability to successfully discriminate stroke patients from this group as well. Consistent with the previous findings, the latency and average velocity emerged as the most significant parameters.

It is noteworthy that the very high success rates may have been partially influenced by the inclusion of healthy individuals in this population. As discussed in the Introduction, this might introduce some degree of bias.

In summary, the multiple analyses presented here convincingly demonstrate that saccade and vergence eye movement parameters can be used to effectively differentiate stroke patients from other populations, including children with learning disorders and individuals with a broad spectrum of dysfunctions or pathologies.

## 4. Discussion

This study demonstrates the existence of several biomarkers in the eye movements of the stroke population, allowing for the screening of stroke cases using eye movement parameters. First, we studied the screening of the stroke population among a group of children and adolescents presenting attention and learning disorders; we achieved a macro F1 score of up to 77.2% when training the random forest on the vergence data. Furthermore, we explored the generalization of our findings to a larger dataset with greater variability in pathologies. We also addressed the discrepancy in the model’s performance in screening stroke and non-stroke cases by applying post hoc calibration, achieving a trade-off with up to 83.8% sensitivity for stroke screening and 68.7% for non-stroke performance. Finally, we tested whether the high scores were due to age bias. By screening the same population using another dataset with age-matched subgroups, we confirmed that the model’s screening ability was preserved. These comparisons confirm that eye movement abnormalities enable us to identify stroke patients among other clinical populations. Next, we will discuss the physiological significance of these results.

### 4.1. General Physiological Discussion

Interest in eye movement in stroke patients has a long history, as it is part of the routine clinical evaluation. However, this evaluation is subjective, with no eye movement recording. The potential of eye tracking to detect and assess neglect is also well documented. Some representative recent studies on the use of eye movement analysis in stroke patients are presented below. Gaol and Sabel [52] reported that hemianopia leads not only to visual field loss but also to enlarged microsaccades and impaired binocular conjugacy, suggesting dysfunction in microsaccade control circuits that worsen over time. However, a microsaccade bias toward the seeing field, indicating the greater allocation of attention, can enhance stimulus detection. The authors concluded that microsaccades may play a compensatory role in visual impairment and provide a foundation for vision restoration and plasticity, warranting further exploration. Cox et al. [53] noted that measuring eye movements in patients with visuospatial neglect is valuable in identifying and understanding additional cognitive impairments, such as in spatial working memory. Hassan et al. [54] reported that automated eye tracking, which can be deployed without calibration, can effectively measure eye movement symmetry and distinguish between normal and abnormal patterns. They emphasized the importance of validating these findings in larger populations. More recently, Ionescu et al. [55] suggested a complex relationship between the cognitive status, gaze fixation behavior, and psychological well-being in stroke patients, advocating for further research with larger sample sizes and a detailed analysis of saccadic eye movements to better understand these relationships and support effective stroke rehabilitation.

The above-cited literature shows the interest in eye movement studies in stroke patients, as well as the limitations of such studies, namely limitations in the types of eye movements studied (microsaccades, saccades only), small numbers of patients, etc. Here, we emphasize that, to detect neglect via eye movement, it is essential to analyze all types of eye movement, particularly saccades and vergence, which are the movements used to explore the 3D environment. In the present study, we did not aim to use eye movements solely to evaluate neglect.

This study was a secondary analysis of large-scale, anonymized time series data obtained from saccade and vergence tests from the Orasis-Ear AIDEAL platform. The AIDEAL software identifies and exports parameters such as the latency, accuracy, velocity, and binocular coordination of eye movements, and these features are used for machine learning. The platform only considers movements that fall within a physiological range, such as those with an amplitude covering at least 50% of the required target amplitude and physiological latencies (e.g., between 90 and 700 ms).

This is particularly important as, in stroke patients (e.g., those with neglect or hemianopsia), eye movements towards the lesioned side can be absent or highly disrupted, with very long latencies and very small amplitudes (such as staircase movements). By considering only movements within well-established physiological limits, we aim to determine whether eye movement function is also impacted on the unaffected side in stroke patients and whether such abnormalities can help to identify stroke patients among other populations.

Thus, abnormalities in the functional and spared visual field of eye movements may serve as a valuable tool in detecting stroke sequelae. Physiologically speaking, we hypothesize that the loss of function in one visual field, whether partial or complete, can still impact eye movements in the spared field, as the eye movement system relies on feedback from the entire visual field to function optimally. To the best of our knowledge, such a quantitative machine learning approach to saccade and vergence eye movements in individuals with a stroke history is completely missing in the literature. We sought to determine whether the motor function per se of saccades and vergence—major movements involved in exploring the environment—was affected by stroke and whether this effect could be distinguished from other clinical pathologies.

The only previous study in a similar context focused on microsaccades [52], but this approach is limited, as microsaccades are difficult to measure and to differentiate from physiological noise. Our study demonstrates that both saccade and vergence eye movements are impacted by stroke, affecting their cortical and subcortical parameters, such as latency and velocity. A delay in eye movement latency suggests cerebral hypofunction due to stroke, while a reduced velocity indicates a brainstem impairment in controlling eye movement execution.

The traditional statistical correlations and machine learning approaches used in the present study both revealed consistent findings regarding latency, velocity, and amplitude issues. The strength of machine learning lies in its ability to consider all parameters simultaneously, providing a more integrative physiological approach. Consequently, the study of data from a large cohort, including approximately 100 stroke patients, reveals specific and subtle eye movement abnormalities in stroke patients’ saccades and vergence, enabling screening among different patient groups. Notably, the reduction in the saccade amplitude in stroke patients aligns with clinically observed restricted and diminished environmental exploration. Even in the preserved visual field, movement can be reduced due to insufficient visual feedback.

The results regarding vergence are intriguing and more complex to interpret. While the initial amplitude may be normal, the response can become unstable, with movements potentially canceled at 80 or 160 ms, particularly for divergence. This instability is likely associated with saccadic intrusions during vergence movements, causing higher instantaneous velocities. This observation suggests an impairment in motor control, i.e., the patient struggles with spatial transition and post-movement fixation stability, sometimes returning to the initial position with uncertainty.

In summary, the dynamics of eye movements, especially when combined with the quantitative power of machine learning, reveal significant insights into brain function and stroke sequelae. The latency and velocity are key features that differentiate stroke patients, indicating that their eye movements are impacted in both the cortical and subcortical regions of the brain. Specifically, the latency is linked to cerebral parietofrontal circuits, while the velocity reflects subcortical eye movement generators in the brainstem. This study offers a comparison with children with neurodevelopmental disorders, who often experience attention issues and neglect, yet stroke patients exhibit more severe disruptions in their eye movement functions. The comparison with an age-matched group further strengthens these findings. Indeed, while the latency is age-dependent, the velocity remains largely unaffected by age, and machine learning approaches benefit from considering multiple features simultaneously. Such eye movement parameters, particularly the latency and velocity, provide sensitive and objective data that could be used to track a patient’s recovery.

### 4.2. Limitations

The present study addresses the need to consider clinical data from various centers to test machine learning models designed to identify a specific pathological group among other patient groups or controls. We believe that this line of research is important and complementary to laboratory research.

It is important to emphasize that all clinical centers used the same methodology to test saccade and vergence eye movements. Moreover, all recordings were obtained using the Pupil Core eye tracker and analyzed with the same software. Both methods are patented. In particular, human factors cannot influence AIDEAL analysis, as it is performed automatically in the cloud. Thus, we believe that the data are well controlled.

A final point concerns the limited, if not non-existent, studies involving objective vergence eye movement recordings in response to unpredictable targets presented at different depths, as achieved with the REMOBI technology. This lack of comparable studies justifies the high rate of self-referencing in our research. Nonetheless, vergence eye movements play a major role in perceiving the 3D visual space.

### 4.3. Future Directions

In this study, we explored the use of machine learning algorithms combined with eye movement parameters to screen for stroke disorders, first among individuals with learning disorders and then within a broader population. Although this approach allows for interpretability, the performance of different models could potentially be optimized by adopting an end-to-end approach. Such an approach would leverage the entire eye movement position series, rather than relying solely on predefined parameters selected by experts.

Another key direction for future research is to assess the generalizability of these findings by using larger and more balanced datasets and performing extensive hyperparamater tuning to increase the performance further.

In this study, we omitted hyperparameter tuning but included a post-processing step to tune the decision threshold. This choice was motivated by the complexity of the two tasks. While threshold tuning involves searching for an optimal threshold within the interval [0, 1], which can be achieved by retaining a small portion of the training samples during each training fold, hyperparameter tuning is an N-dimensional optimization problem, requiring substantially more data.

As a result, although hyperparameter tuning could improve the model performance, it may not be relevant when working with small datasets, as it would require an additional validation set on top of the training and testing sets. A future direction could be to optimize these methods further using larger datasets.

## 5. Conclusions

In conclusion, the present study combined the binocular eye movement analysis of saccades and vergence with machine learning, introducing new tools to examine brain function in stroke patients that is more nuanced than gross or bedside clinical evaluations. This study demonstrates that eye movement parameters, particularly the latency and average velocity, can effectively differentiate stroke patients from a wide range of other clinical populations, with the random forest model showing the best overall performance for this task. Ongoing studies could develop further AI approaches including deep learning. Such knowledge could ultimately be translated into an AI-based assistant for the monitoring of sequelae years after a stroke or for the evaluation of progress before and after treatment. Such refined tools could provide a deeper understanding of the ongoing challenges faced by patients in tasks requiring close-range control, such as reading, years after a stroke.

## 6. Patents

Zoï Kapoula has applied for patents for the technology used to conduct this experiment.

- REMOBI is an embedded device with many paradigms to stimulate and record saccade, vergence, and combined eye movements in 3D space (patent US8851669, WO2011073288), REMOBI Neuro Cog, EP 4 215 172 A1.
- AIDEAL is an eye movement analysis program, WO EP US WO2021228724A; a pending patent application concerns learning disorders, EP21798945.8A OWO 2021/228724 A1WO2021228724A1 (https://patentimages.storage.googleapis.com/a2/36/b6/e9d7ca92f21cc7/WO2021228724A1.pdf, accessed on 17 February 2025).

REMOBI table (patent US8851669, WO2011073288); AIDEAL (Artificial Intelligence Eye Movement) Analysis (EP20306166.8, 7 October 2020; EP20306164.3, 7 October 2020—Europe). Patent application pending EP22305903.1.

## Figures and Tables

**Table 1:** Presentation of the mean age (in years), standard deviation, and total number of patients for each of the stroke and non-stroke groups and for each of the non-stroke datasets.

Group	Mean	Std	Number of Patients
Stroke (Datasets 1 and 2)	48.45	23.31	82
Learning Disorder	13.63	7.78	475
Global Clinical Population	16.0	17.96	1280
Age-Matched Group	54.3	6.67	99

**Table 2:** Number of eye movement recordings for stroke and non-stroke individuals available in the two datasets. In Dataset 1 (stroke vs. learning disorders), the non-stroke group includes recordings annotated as learning disorders. In contrast, in Dataset 2 (stroke vs. global clinical population), the non-stroke group consists of all recordings that lack the neurological stroke annotation.

Dataset	Saccade			Vergence		
	Stroke	Non-Stroke	Imb.	Stroke	Non-Stroke	Imb.
Dataset 1	167	488	2.92	105	562	5.35
Dataset 2	167	1256	7.52	105	1573	14.98

**Table 3:** Mean values for each of the three groups along with the Kruskal–Wallis statistical test used to evaluate the significance of comparisons with the stroke population when performing the saccade eye movement test. Note that *, **, and *** indicate *p*-values (after Bonferroni correction) of less than 0.016, 0.0033, and 0.0003, respectively, for all eye movement parameters except the amplitude, duration, and maximum velocity. For these three parameters, the significance thresholds were adjusted by dividing the *p*-values by 2, resulting in thresholds of 0.025, 0.005, and 0.0005, respectively.

Parameter		Stroke	Learning Disorder		Global Clinical Population	
		Mean	Mean	Stat	Mean	Stat
Left	Amplitude	11.33	12.18	21.49 ***	12	16.95 ***
	Latency	269.97	233.78	12.74 **	223.97	24.58 ***
	Duration	59.89	61.29	6.17 *	61.23	4.93
	Max. Vel.	292.69	311.49	4.96	306.11	3.23
	Avg. Vel.	151.6	174.95	6.26 *	179.48	9.6 **
	Drift 1	0.68	0.64	2.4	0.57	4.05 *
	Drift 2	0.75	0.88	0.82	0.78	1.09
	Sacc. Disc.	2.9	2.58	0.94	2.79	0.04
Right	Amplitude	11.55	12.1	8.89 **	11.96	6.46 *
	Latency	276.67	217.76	19.89 ***	214.22	27.88 ***
	Duration	59.11	60.62	2.67	59.58	1.53
	Max Vel.	326.02	315.98	1.23	307.27	0.85
	Avg. Vel.	155.46	184.4	11.07 **	187.33	14.52 ***
	Drift 1	0.53	0.68	0.88	0.6	0.53
	Drift 2	0.7	0.89	0.47	0.82	0.31
	Sacc. Disc.	3.2	2.67	6.07 *	2.9	1.08

The bold highlights the statistical significant difference between the two groups for the corresponding parameters.

**Table 4:** Group means for each of the three groups together with the Kruskal–Wallis test value for comparisons between the stroke population and the learning disorder population and between the stroke population and the global clinical population. Note that *, **, and *** indicate *p*-values (after Bonferroni correction) of less than 0.016, 0.0033, and 0.0003, respectively, for the latency, phasic amplitude, average velocity, and average phasic velocity. For the remaining parameters, the significance thresholds were adjusted by dividing the *p*-values by 2, resulting in thresholds of 0.025, 0.005, and 0.0005, respectively.

Parameter		Stroke	Learning Disorder		Global Clinical Population	
		Mean	Mean	Stat	Mean	Stat
**Conv.**	Phasic Amplitude	3.27	3.08	0.93	3.4	0.09
	Latency (ms)	358.35	349.12	2.31	316.81	22.07 ***
	Duration (ms)	63.27	69.53	1.57	71.47	3.52
	Max. Vel. (°/s)	200.55	119.08	9.79 **	117.94	11.31 **
	Avg. Vel. (°/s)	71.11	47.42	25.46 ***	49.71	21.19 ***
	Ampl. (80 ms) (°)	0.62	0.85	10.2 **	0.89	16.59 ***
	Ampl. (160 ms) (°)	0.97	1.44	16.08 ***	1.41	21.4 ***
	Phas. Ampl. (160 ms)	4.25	4.43	0.10	4.71	2.48
	Phas. Avg. Vel. (°/s)	19.05	19.59	0.11	20.5	1.89
**Div.**	Phasic Amplitude	3.19	2.59	16.49 ***	2.63	17.35 ***
	Latency (ms)	364.66	345.16	4.54 *	327.36	16.64 ***
	Duration (ms)	65.42	67.60	0.05	68.58	0.07
	Max. Vel. (°/s)	148.49	97.98	13.94 ***	104.26	13.48 ***
	Avg. Vel. (°/s)	52.87	39.53	6.24 *	42.85	5.96 *
	Ampl. (80 ms) (°)	0.59	0.66	5.66 *	1.04	15.27 ***
	Ampl. (160 ms) (°)	0.92	1.13	14.13 **	1.55	25.27 ***
	Phas. Ampl.	3.90	3.57	1.55	3.98	0.94
	Phas. Avg. Vel. (°/s)	17.41	16.02	2.42	17.99	1.59

The bold highlights the statistical significant difference between the two groups for the corresponding parameters.

**Table 5:** Performance of three models in various metrics for saccade and vergence tests when screening for stroke among those with learning disorders: macro F1, accuracy, sensitivity, and specificity scores.

Model	Saccade				Vergence			
	F1	Acc.	Sens.	Spec.	F1	Acc.	Sens.	Spec.
Logistic Regression	60.0%	67.0%	49.0%	73.0%	59.5%	69.9%	60.9%	71.5%
SVM (RBF)	70.2%	75.4%	65.9%	78.7%	62.4%	74.1%	58.1%	77.0%
Random Forest	**74.1%**	83.5%	45.5%	96.5%	**77.2%**	90.7%	43.8%	99.5%

The bold indicates the best models in term of F1 score.

**Table 6:** Performance of three models in various metrics in the test set of each fold for the screening of stroke among the global clinical population: macro F1, accuracy, sensitivity, and specificity scores.

Model	Saccade				Vergence			
	F1	Acc.	Sens.	Spec.	F1	Acc.	Sens.	Spec.
Logistic Regression	54.6%	70.5%	48.5%	73.4%	54.2%	72.3%	75.2%	72.1%
SVM (RBF)	61.5%	78.8%	50.3%	82.6%	55.2%	76.8%	59.0%	77.9%
Random Forest	**75.6%**	92.7%	38.3%	99.9%	**77.1%**	96.2%	39.0%	100.0%

The bold indicates the best models in term of F1 score.

**Table 7:** Performance metrics for random forest (RF) models with and without threshold tuning. Metrics include F1 score (F1), accuracy (Acc.), sensitivity (Sens.), and specificity (Spec.).

Model	Saccade				Vergence			
	F1	Acc.	Sens.	Spec.	F1	Acc.	Sens.	Spec.
RF Without Threshold Tuning	75.6%	92.7%	38.3%	99.9%	77.1%	96.1%	39.0%	100.0%
RF With Threshold Tuning	62.4%	74.3%	76.6%	74.0%	53.4%	69.7%	83.8%	68.7%

**Table 8:** Performance metrics for logistic regression, SVM (RBF), and random forest models. Metrics include F1 score (F1), accuracy (Acc.), sensitivity (Sens.), and specificity (Spec.) for both saccades and vergence.

Model	Saccade				Vergence			
	F1	Acc.	Sens.	Spec.	F1	Acc.	Sens.	Spec.
Logistic Regression	89.2%	89.7%	87.4%	93.7%	90.5%	90.6%	89.5%	91.9%
SVM (RBF)	87.35%	87.8%	84.4%	93.7%	91.0%	91.0%	90.4%	91.9%
Random Forest	93.7%	94.3%	98.8%	86.4%	90.4%	90.6%	94.3%	86.0%

**Figure A6:**
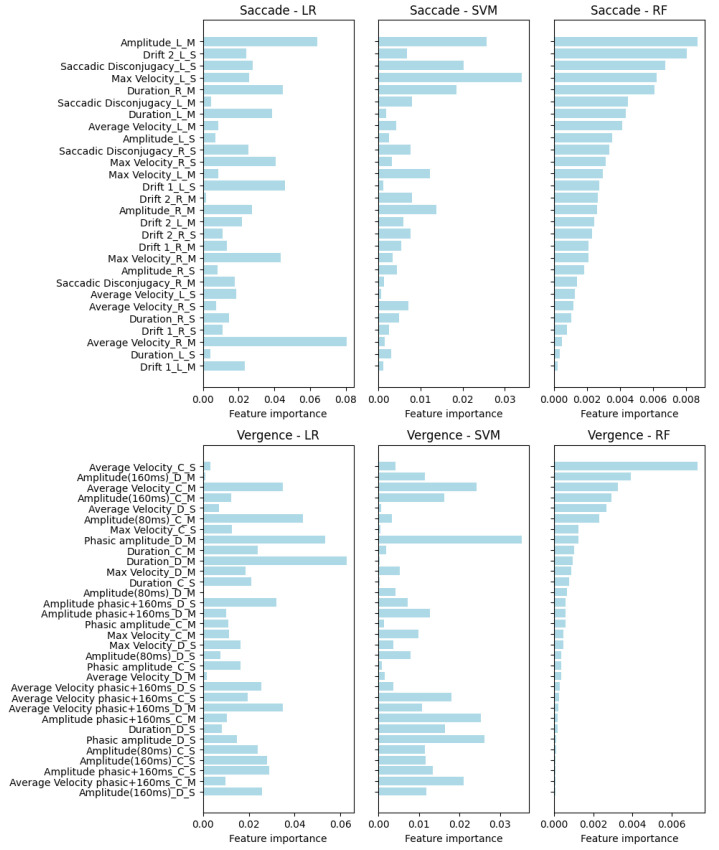
The feature importance for each dataset and for each algorithm when screening for stroke among all other non-stroke populations.

## Data Availability

The datasets generated during and/or analyzed during the current study are not publicly available due to privacy. However, upon reasonable request, they can be made available by the corresponding author.

## Appendix B

**Table A1:** Definitions of the 8 acronyms used to abbreviate the eye movement parameters.

Acronym	Definition
RM (CM)	Right Mean (Convergent Mean)
LM (DM)	Left Mean (Divergent Mean)
RS (CS)	Right Standard Deviation (Convergent Std)
LS (DS)	Left Standard Deviation (Divergent Std)

**Table A2:** The set of eye movement parameters computed by the software AIDEAL for each trial, depending on the type of test.

Saccade Analysis	Vergence Analysis
Amplitude	Phasic Amplitude
Latency	Latency
Duration	Duration
Max. Velocity	Max Vel.
Avg. Velocity	Avg. Vel.
Drift 1	Amplitude (80 ms)
Drift 2	Amplitude (160 ms)
Saccadic Disconjugacy	Amplitude Phasic
	Average Velocity (Phasic + 160 ms)

## Appendix C

**Table A3:** Mean values for the stroke and Martin datasets, along with the statistical test results for significance comparisons when performing the saccade eye movement test. Note that **, and *** indicate *p*-values of less than 0.01 and 0.001, respectively.

Parameter		Stroke (Mean)	Martin Dataset (Mean)	Stat
Left	Latency	269.97	271.56	0.99
	Avg. Vel.	151.6	267.21	103.96 ***
	Drift 1	0.68	0.64	26.63 ***
	Drift 2	0.75	0.74	9.6 **
	Sacc. Disc.	2.9	1.85	4.11
Right	Latency	276.67	281.03	2.97
	Avg. Vel.	155.46	264.84	96.02 ***
	Drift 1	0.53	0.67	17.09 ***
	Drift 2	0.7	0.78	5.33
	Sacc. Disc.	3.2	2.0	2.22

**Table A4:** Mean values for the stroke and Martin datasets, along with the statistical test results for significance comparisons when performing the saccade and vergence eye movement tests. Note that * and ** indicate *p*-values of less than 0.05 and 0.01, respectively.

Parameter		Stroke (Mean)	Martin Dataset (Mean)	Stat
Conv.	Latency	358.35	340.18	5.56
	Avg. Vel.	71.11	45.08	9.94 **
	Ampl. Phasic	4.25	5.05	7.64 *
	Avg. Vel. Phasic	19.05	20.95	6.42 *
Div.	Latency	364.66	349.84	1.74
	Avg. Vel.	52.87	41.49	0
	Ampl. Phasic	3.9	3.46	1.06
	Avg. Vel. Phasic	17.41	16.63	0.07
